# Unraveling the Impacts of Long-Term Exposure in Low Environmental Concentrations of Antibiotics on the Growth and Development of *Aquatica leii* (Coleoptera: Lampyridae) from Transcription and Metabolism

**DOI:** 10.3390/insects16121239

**Published:** 2025-12-08

**Authors:** Jiapeng Li, Jiani Lin, Chao Liu, Lihong Yan, Qimeng Wang, Zitong Zhou, He Lv, Chengquan Cao, Yiping Wang

**Affiliations:** 1 College of Forestry and Biotechnology, Zhejiang Agricultural and Forestry University, Hangzhou 311300, China; lijiapeng2024@stu.zafu.edu.cn (J.L.); 13738327890@163.com (J.L.); wa88711007@163.com (C.L.); yanlihong2023@163.com (L.Y.); wqm010405@163.com (Q.W.);; 2 College of Life Sciences, Huzhou University, Huzhou 313000, China; 02592@zjhu.edu.cn; 3 College of Life Sciences, Leshan Normal University, Leshan 614004, China; chqcao1314@163.com

**Keywords:** *Aquatica leii*, transcriptome, metabolome, antibiotics, oxytetracycline, levofloxacin

## Abstract

Widely used in humans and animals for bacterial infection management, antibiotics are poorly absorbed and persistent, being excreted into ecosystems and causing serious pollution. However, the effects of extended exposure to residual environmental levels of antibiotics in fireflies are currently unknown. In this study, we investigated *Aquatica leii* growth and development under chronic low-dose exposure to oxytetracycline (OTC) and levofloxacin (LEV) residues using physiology, transcriptomics, and metabolomics. The results showed that OTC and LEV treatments enhanced *A. leii* larval growth (increased body length and weight) and significantly altered SOD and CAT activities. Transcriptional analyses identified 1193 and 1669 differentially expressed genes from the LEV and OTC groups, respectively, compared to the control group, predominantly associated with insect hormone biosynthesis, digestive system, immune system, and signal transduction. The metabolomic results showed that 965 and 1094 endogenous metabolites (i.e., comprising mainly amino acids, organic acids, and carbohydrates) were affected by LEV and OTC treatments, respectively. These metabolites are mainly enriched in signal transduction, amino acid metabolism, and energy metabolism pathways. Further, a conjoint omics analysis highlighted the multifaceted regulatory roles of glutathione metabolism in the response of *A. leii* to OTC and LEV treatments. The study provides critical insights into the transcriptional and metabolic responses of *A. leii* to antibiotic treatment, informing dosage guidelines for sustainable *A. leii* farming.

## Simple Summary

Widely used in humans and livestock, antibiotics are poorly absorbed and persistent, causing serious pollution through excretion into ecosystems. Fireflies are an important indicator species of this environmental change, which are particularly sensitive to water contamination. However, whether environmental concentrations of antibiotics pose a hazard to fireflies remains an unexplored area of research. In this study, we investigated the growth and development of *Aquatica leii* larvae under chronic low-concentration oxytetracycline (OTC) or levofloxacin (LEV) exposure using integrated physiological, transcriptomic, and metabolomic analyses. Our research found that *A. leii* larvae in the OTC and LEV treatment groups exhibited increased body length and weight, along with significantly altered superoxide dismutase (SOD) and Catalase (CAT) activities. In addition, there were significant changes in gene expression in *A. leii* in the OTC and LEV groups, involving key physiological processes such as insect hormone biosynthesis digestive system, immune system, and signal transduction. Similarly, endogenous metabolites were affected by LEV and OTC treatments. These metabolites are mainly enriched in signal transduction, amino acid metabolism, and energy metabolism pathways. Our results will help to understand the molecular mechanisms of *A. leii* response to antibiotic treatment and provide a theoretical reference for the usage and dosage of antibiotics in *A. leii* farming.

## 1. Introduction

Fireflies (Coleoptera: Lampyridae) are considered to be among the most beautiful insects in the world because of their unique bioluminescent courtship display [1,2]. Each year, tens of thousands of visitors come to appreciate the ecological landscape of the flashing light behavior of fireflies in firefly parks or natural habitats, thereby providing enormous social and economic benefits through local ecotourism [3,4]. However, over the past few years, firefly populations have been experiencing rapid declines due to habitat loss and degradation, artificial light disrupting courtship communication, indiscriminate use and mishandling of synthetic pesticides, and anthropogenic climate change [5,6]. The aquatic firefly *Aquatica leii* is a species exclusively found in China that is grown in aquaculture ponds and rice paddy fields [7]. Freshwater pollution is often considered as a major threat to aquatic habitats [8]. Consequently, the occurrence of antibiotics in water bodies is considered an emerging threat to biodiversity and ecosystem functioning [9]. It is suggested that the consumption of antibiotics surged by approximately 69% between 2000 and 2015, with an average annual increase of more than 4% [10]. Without preventive interventions or mitigation measures, a threefold increase in global antibiotic use is projected during the 2015–2030 timeframe [11]. Through continuous input from human-related activities, antibiotics accumulate in aquatic habitats, leading to sustained, low-dose exposure among aquatic biota, which has the potential to impact aquatic ecosystems [12,13].

Antibiotics are drugs, either natural or synthetic, that are utilized to manage bacterial infections by killing bacteria or halting their growth [14]. Beyond that, antibiotics have seen broad usage in the livestock industry as a means of controlling infections and improving feed efficiency, in aquaculture industries and insect farming to effectively control mortality and morbidity, and to support improved weight gain and feed efficiency [15,16,17,18]. Nevertheless, due to limited intestinal absorption, a large amount of administered antibiotics is excreted unchanged, entering natural ecosystems through animal waste [12,19]. Other studies demonstrated that environmental pollution caused by antibiotics may pose adverse effects to hydrobiota [17,20]. For example, previous studies revealed that regardless of the exposure route, chronic low environmental concentrations of antibiotics severely impair *Oreochromis niloticus* health by disrupting general physiological functions and nutritional metabolism and compromising their immune systems [12]. Antibiotics, even at environmentally relevant concentrations, do not exhibit acute toxicity but can still have sublethal effects on teleosts and benthic macroinvertebrates [21,22,23]. Depending on the severity, sublethal impacts can occur in fish, such as oxidative stress, metabolic disorders, histological damage, neurotoxicity, malformations, and genotoxicity [12,21,24]. In aquatic invertebrates, besides oxidative stress, alterations in growth and development, reduced reproductive capacity, and impacts on energy metabolism were noted as well [21,25].

Oxytetracycline (OTC) is one of the tetracycline antibiotics, which inhibits bacterial growth by binding to the 30S ribosomal subunit, thereby preventing the binding of aminoacyl-tRNA to the ribosome [12,26]. Levofloxacin (LEV) is a fluoroquinolone antibiotic that inhibits bacterial DNA gyrase, thereby precluding the synthesis of functional bacterial DNA and impairing bacterial viability and inducing cell death [27]. Due to their potent antimicrobial activity and minimal side effects, LEV and OTC serve as commonly applied feed additives in both terrestrial and aquatic animal farming practices [28]. However, these drugs inevitably enter aquatic environments. Numerous studies have reported OTC concentrations of 15,163 ng/L in seawater and a concentration range of 174.9 ± 266.9 ng/L to 741.85 ng/L among aquatic habitats [12,29,30]. Similarly, LEV has been frequently detected in various riverine systems, with concentrations exceeding permissible levels: Japan (3600 ng/L), China (6800 ng/L), Portugal (34–438 ng/L), and India (2030 ng/L) [31,32]. In summary, these antibiotic contaminants exhibiting high detection frequencies and concentrations have attracted widespread attention.

The aquatic firefly *A. leii*, widely distributed in streams, lakes, and paddy fields, serves as a key indicator of environmental conditions in freshwater ecosystems [6]. However, different classes of antibiotics frequently coexist in the natural environment due to overlapping sources of contamination [33]. Although the toxicity of antibiotics to fish, shrimp, and algae growth was explored by many researchers, the toxicity of antibiotics to fireflies has been overlooked [16,34,35]. Therefore, we aim to investigate chronic low-dose exposure levels regarding the impacts of antibiotics on the freshwater firefly, *A. leii*. In this study, we reared *A. leii* larvae in environments with low concentrations of LEV and OTC to explore the impacts of these two antibiotics with implications for growth and development, antioxidant enzyme activity, gene transcription, and metabolism of the *A. leii*. This research may elucidate the effects of antibiotics on *A. leii*, thereby deepening our understanding of the survival capacity of aquatic invertebrates under low environmental concentrations of antibiotics. By integrating theoretical frameworks with applied research, this study provides a scientific reference for optimizing antibiotic use in the artificial breeding of *A. leii*.

## 2. Materials and Methods

### 2.1. Insect Materials

Eggs of *A. leii* were obtained from the Qingying Firefly Conservation and Restoration Center located in Qionglai City, Chengdu, Sichuan Province, and were hatched in the laboratory of Zhejiang Agricultural and Forestry University, Zhejiang, China. Eggs were maintained at 25 ± 1 °C and 90% humidity and turned once every eight hours for the duration of the incubation period. Soon after hatching, 3000 first-instar larvae were transferred to six separate containers, each measuring 25 × 15 × 10 cm and containing 1 L of well-dechlorinated tap water. The larvae rearing conditions were maintained at 25 ± 1 °C, pH of 6.5~7.5, and dissolved oxygen content of 7.0 ± 0.3 mg/L. *A. leii* larvae were fed chironomid larvae and *Cipangopaludina chinensis* on a bi-daily schedule, as described previously [7].

### 2.2. Antibiotics Treatment and Sample Collection

Oxytetracycline (OTC; Sangon Biotech, Shanghai, China; NO. A430183) and levofloxacin (LEV; Sangon Biotech; NO. A358214) were diluted to a concentration of 50 mM in DMSO and stored at –20 °C. Two days after hatching, first-instar larvae of *A. leii* were independently exposed to water containing 420 ng/L and 250 ng/L of OTC and LEV. During the 160-day exposure, we performed complete water renewal every 24 h with freshly prepared test solutions to maintain nominal concentrations. The final DMSO concentration was below 1% *v*/*v* (volume-to-volume ratio) in both the test and control groups throughout the experiment. These two concentrations were selected to mimic low, environmentally representative exposure scenarios according to prior results [12,36]. Moreover, we maintained a control group and established triplicate replicates for each treatment. The experiment lasted for 160 days, and the weight and body length were recorded once per instar. After reaching the sixth age stage, 30 individual *A. leii* larvae were obtained from each group in isolation from the OTC and LEV treatments and the control group. Biological triplicates were maintained for each treatment condition, and for each replicate, 10 larvae were pooled together to reduce individual variability, as described previously [7]. While this approach sacrifices individual-level resolution, it is statistically and practically justified for this species. Samples were immediately immersed in liquid nitrogen to preserve molecular integrity and stored at −80 °C for downstream analysis. The animal utilization protocol was approved by the Institutional Animal Care and Use Committee of Zhejiang Agricultural and Forestry University.

### 2.3. Antioxidant Enzyme Activity Assays

The activity of superoxide dismutase (SOD) was examined by the nitroblue tetrazolium (NBT) method with an optical density at 560 nm via a spectrophotometric assay [37]. Catalase (CAT) activity was determined by using a catalase (CAT) activity assay kit, following the manufacturer’s recommendations. All experiments were independently repeated three times. The SOD activity detection kit (cargo number: BC0205) and CAT assay kit (cargo number: BC5165) were sourced from Beijing Solabio Life Sciences Co., Ltd., Beijing, China.

### 2.4. RNA Isolation and Transcriptome Sequencing

Total RNA was isolated using TRIzol™ Reagent (Invitrogen Life Technologies, Carlsbad, CA, USA), as per the manufacturer’s recommendations. RNA integrity and removal of genomic DNA were obtained by 1.5% agarose gel electrophoresis, as described previously [7]. Subsequently, the concentration, quality, and integrity of the RNA were determined via a Thermo Scientific NanoDrop 2000 (Thermo Scientific, Waltham, MA, USA), a Qubit2.0 Fluorometer (Thermo Scientific, Waltham, MA, USA), and an Agilent 2100 Bioanalyzer (Agilent Technologies, Santa Clara, CA, USA). Sequencing libraries were derived from 1 µg of total RNA using the NEBNext Ultra II DNA Library Prep Kit for Illumina (New England Biolabs, Ipswich, MA, USA) and were sequenced to generate 150 bp paired-end reads on the Illumina Novaseq 6000 platform by Shanghai Personal Biotechnology Co., Ltd., Shanghai, China

### 2.5. Transcriptome Assembly, DEG Screening, and Enrichment Analyses

Raw sequencing reads were subjected to quality trimming using the fastp (v.0.22.0) [38] software for excluding substandard values and adapter sequences based on Zhou et al. [39]. The filtered reads were aligned to the reference genome of *A. leii* (NCBI accession number: GCA_035610365.1) [1] using HISAT2 (v.2.1.0) [40], and the mapped reads were obtained via StringTie [41] with the *A. leii* genome. The original expression of the genes was quantified via HTSeq (v0.9.1) [42], and then the expression values were standardized using fragments per kilobase of transcript per million mapped fragments (FPKM) by the RSEM software (v.1.3.3) [43]. Significantly differentially expressed genes (DEGs) were analyzed by DESeq2 (v1.38.3) [44] with the following screening conditions: |log2FoldChange| > 1.0 and adjusted *p*-value < 0.05. Using the Gene Ontology (GO) database, genes were mapped to functional terms, and the abundance of DEGs within each GO category was computed. Then, we performed KEGG analyses on these DEGs using the EnrichKEGG functions in clusterProfiler (v4.6.0) and calculated the adjusted *p*-values via the hypergeometric distribution method [45]. Transcriptome analysis data have been published, and the NCBI accession number is PRJNA1372381.

### 2.6. Metabolite Extraction and Detection

Approximately 30 mg of *A. leii* larvae from each replicate within the OTC and LEV exposure groups was sampled independently. Metabolites were retrieved from the larvae using the water, methanol–acetonitrile (*v*/*v* 1:1), and 2-chlorophenylalanine (5 ppm) methanol solution protocol according to previous findings [37,46]. Quality control was performed using 20 μL aliquots from each sample, and the rest was examined via LC-MS. Using an ACQUITY UPLC I-Class system equipped with an ACQUITY UPLC HSS T3 column (2.1 × 150 mm, 1.8 µm) (Waters, Milford, MA, USA), the liquid chromatography (LC) analysis was conducted. Subsequently, mass spectrometry analysis was carried out via a Thermo Orbitrap Exploris 120 mass spectrometer (Thermo Fisher Scientific, Bremen, Germany) using the Xcalibur 4.7 (Thermo Scientific, Waltham, MA, USA) software in both positive and negative ion modes in a data-dependent acquisition (DDA) manner, according to prior research [37,47]. Metabolome raw data have been published, and the National Genomics Data Center (NGDC) accession number is PRJCA052586.

### 2.7. Metabolome Analysis

LC-MS raw data were processed for peak alignment, baseline correction, and missing data imputation, which were performed to standardize the chromatographic profiles across all samples and normalization using the MS-DIAL software version 4.9.221218 (Yokohama, Japan) [48]. Identification of metabolites was achieved by matching MS data against the PerSonalbio Next-Generation Metabolomics Database, which comprises standard product self-built databases, the mzcloud library (https://www.mzcloud.org/ accessed on 17 April 2025), HMDB (http://www.hmdb.ca/ accessed on 17 April 2025) and LIPID MAPS (https://www.lipidmaps.org/ accessed on 17 April 2025), MoNA (https://mona.fiehnlab.ucdavis.edu accessed on 17 April 2025), NIST_2020_MSMS, and an AI-predicted MSMS spectral library. With the R package ropls (version V1.22.0), we performed principal component analysis (PCA) and orthogonal partial least squares discriminant analysis (OPLS-DA), as previously described [37]. Annotation confidence levels for each metabolite were provided, based on the Metabolomics Standards Initiative (MSI) confidence scheme, and the annotation confidence levels are noted in Table S1. Using variable importance projection (VIP) values from the OPLS-DA analysis, we identified differentially accumulated metabolites (DAMs) when VIP > 1, |log2(FC)| > 1 and *p* < 0.05 according to a previous study [49]. Subsequently, we utilized the KEGG database to analyze the biological roles and metabolic pathways of the detected metabolites and visualized pathway enrichment through bubble plots.

### 2.8. Combination Analysis

Additionally, the KEGG database was also used to identify genes corresponding to metabolite-related enzymes based on gene and metabolite information. The differential trends of metabolites and genes with corresponding relationships were organized, and an analysis of common pathways enriched by differences between the two omics was conducted. Co-annotated pathway diagrams for metabolites and genes were simultaneously generated.

### 2.9. Statistical Analysis

One-way ANOVA with Tukey’s multiple comparison test was conducted on all data using Data Processing System (DPS version v21.05, Zhejiang, China). Data are shown as mean ± SD, with *p* < 0.05.

## 3. Results

### 3.1. Influence of OTC and LEV on Growth, Development, and Physiology of A. leii

**Figure 1:**
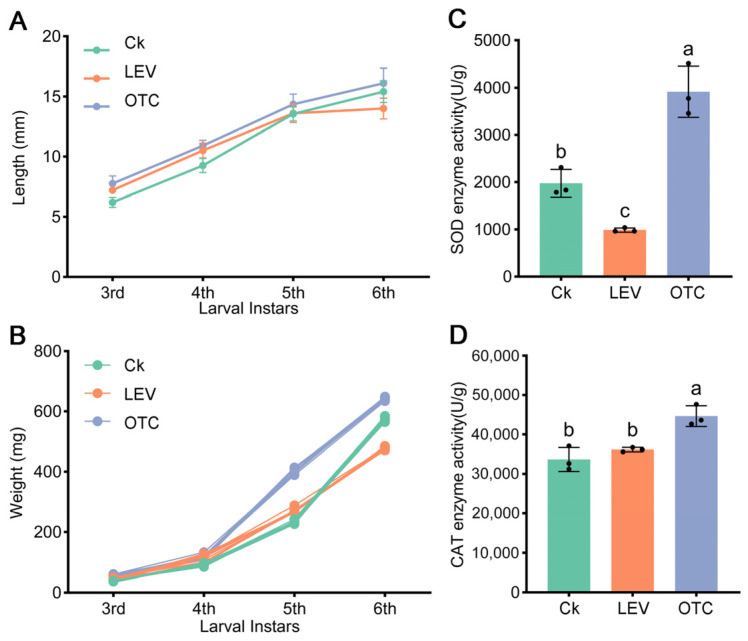
Effect of OTC and LEV treatments on body length, body weight, and physiology of *A. leii*. (**A**) Body length and (**B**) body weight at for each instar at OTC and LEV treatments. Each point represents mean, error bars indicate SD, and each point represents an individual animal (*n* > 10). Enzyme activities of (**C**) SOD and (**D**) CAT. Lowercase letters indicate significant differences from Tukey’s test (*p* < 0.05); error bars indicate SD.

To explore the impacts of low environmental concentrations of OTC and LEV on the growth, development, and physiology of *A. leii* larvae, we first measured their body length and weight throughout the entire larval stage (Figure 1). We found an interesting phenomenon where *A. leii* larvae in the OTC and LEV treatments groups showed larger values of body length and body weight than the control group before the fifth instar phase (Figure 1A,B and Table S2). Then, upon reaching the sixth instar, OTC remained superior to the control group in both body length and body weight, whereas LEV fell below the control group (Figure 1A,B and Table S2). In addition, the activities of SOD and CAT in *A. leii* sixth-instar larvae exposed to OTC and LEV were obtained. As shown in Figure 1C,D, the OTC treatment significantly increased SOD and CAT levels in the sixth-instar larvae of *A. leii*. However, SOD activity was significantly decreased in the sixth-instar larvae of *A. leii* treated with LEV, whereas CAT activity showed no significant change (Table S3).

### 3.2. Transcriptomic Changes Under OTC and LEV Treatments of A. leii

For investigating the underlying biochemical and genetic mechanisms of *A. leii* in response to OTC and LEV exposure, we performed RNA-Seq on sixth-instar larvae of *A. leii* that had been incubated in OTC and LEV for a long time. As shown in Table S3, we acquired an average of 48.98 million clean reads after quality control for raw sequencing reads, with an average mapping rate to the *A. leii* genome of 81.83%. Average Q20 and Q30 quality scores were approximately 98.27% and 96.99%, respectively (Table S3). The Pearson correlation coefficient indicated that intra-group samples exhibited higher correlations and were more clustered compared with intergroup samples (Figure S1A). Again, the results of the principal component analysis indicate that replicate samples within each sample group clustered together (Figure S1B). The above results suggest that there was good analytical stability and experimental reproducibility for this research.

**Figure 2:**
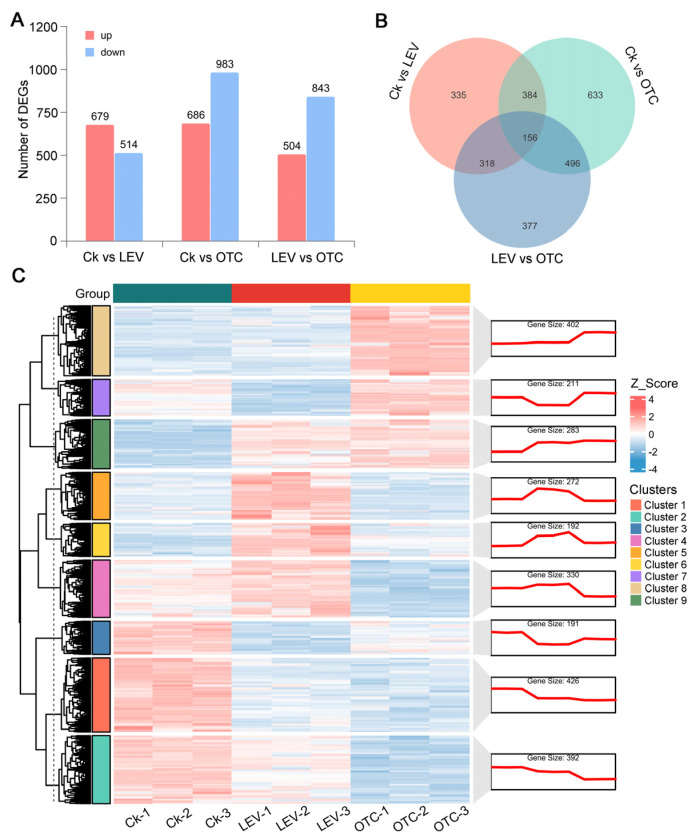
Transcriptomic changes under OTC and LEV treatments in *A. leii*. (**A**) Numbers of the upregulated and downregulated DEGs in *A. leii* OTC and LEV treatments. (**B**) Intersection Venn diagram showing the DEGs between Ck vs. OTC, Ck vs. LEV, and LEV vs. OTC. (**C**) Heatmaps of expression profiles and expression trend maps of DEGs detected in *A. leii* after OTC and LEV treatments.

Then, DEGs were detected in *A. leii* under the OTC and LEV treatments. For Ck vs. LEV, this study annotated 1193 DEGs, with 679 upregulated and 514 downregulated DEGs. A total of 1669 DEGs (686 upregulated and 983 downregulated genes) were recognized at Ck vs. OTC (Figure 2A). Additionally, to better understand the DEGs involved in responses to OTC and LEV treatments, we examined the DEGs that were both shared and unique under these treatment conditions (Figure 2B). It was found that 384 genes were common with both LEV-induced genes and OTC-induced genes, and 1195 genes were uniquely induced by OTC. Overall, the OTC treatment caused an enhanced transcriptional response in *A. leii* when compared with the LEV treatment (Figure 2B). To further explore expression patterns, a heatmap was employed for hierarchical cluster analysis of DEGs, which showed significant differences in *A. leii* following the OTC and LEV treatments (Figure 2C). Then, DEGs were grouped into different clusters. In detail, Clusters 7, 8, and 9 included 896 genes that increased under the OTC treatment; Clusters 4, 5, and 6 included 796 genes that increased under the LEV treatment group; and Clusters 1, 2, and VII3 included 1009 genes that decreased during the the OTC and LEV treatments (Figure 2C). Gene expression analyses imply that *A. leii* larvae responded to the antibiotic treatments in a treatment-specific manner.

### 3.3. KEGG Enrichment Analysis of the LEV and OTC-Responsive DEGs of A. leii

**Figure 3:**
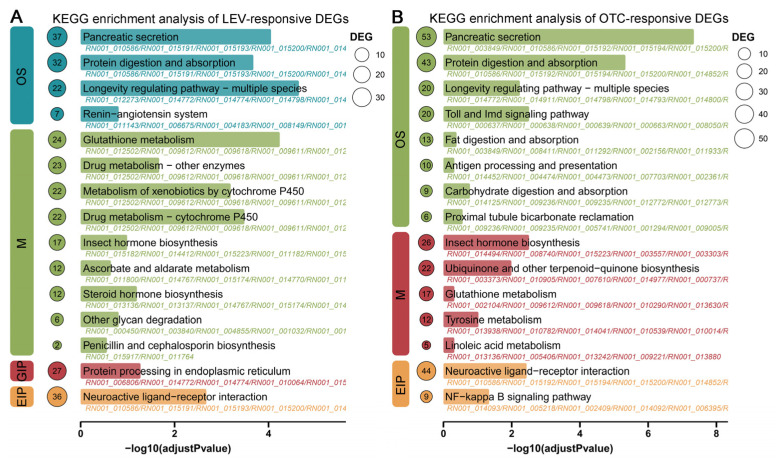
Enrichment analysis of KEGG pathways for DEGs in *A. leii* under OTC and LEV treatments using bar charts. (**A**) Top 15 enriched KEGG pathways of DEGs under LEV treatment. (**B**) Top 15 enriched KEGG pathways of DEGs under OTC treatment. Different colors represent different categories. OS (Organismal Systems), M (Metabolism), GIP (Genetic Information Processing), and EIP (Environmental Information Processing). The size of the dot indicates the number of DEGs.

For exploring the adaptive strategy of *A. leii* to LEV and OTC, this study analyzed the KEGG pathway enrichment of DEGs responsive to the LEV and OTC treatments, employing Fisher’s exact test to determine *p*-values and adjusted via Bonferroni’s correction for each KEGG pathway (Figure 3). In Organismal Systems pathways, the pathways “Pancreatic secretion” “Protein digestion and absorption”, and “Longevity regulating pathway—multiple species” were significantly enriched during the OTC and LEV treatments. In Environmental Information Processing pathways, the pathway “Neuroactive ligand-receptor interaction” was significantly enriched following the LEV and OTC treatments. For Metabolism pathways, the pathways “Glutathione metabolism” “Drug metabolism—cytochrome P450” “Metabolism of xenobiotics by cytochrome P450” “Drug metabolism—other enzymes” “Steroid hormone biosynthesis”, and “Insect hormone biosynthesis” were significantly enriched following the LEV treatment; the pathways “Insect hormone biosynthesis” and “Ubiquinone and other terpenoid-quinone biosynthesis” were significantly enriched following the OTC treatment (Figure 3, Tables S4 and S5). Among them, “Insect hormone biosynthesis” was the only common metabolism pathway to the OTC and LEV groups, indicating that it was probably the most important pathway involved in responses to antibiotic treatment in *A. leii*.

### 3.4. Differential Expression of Insect Hormone-Biosynthesis-Related Genes Under OTC and LEV Treatments

**Figure 4:**
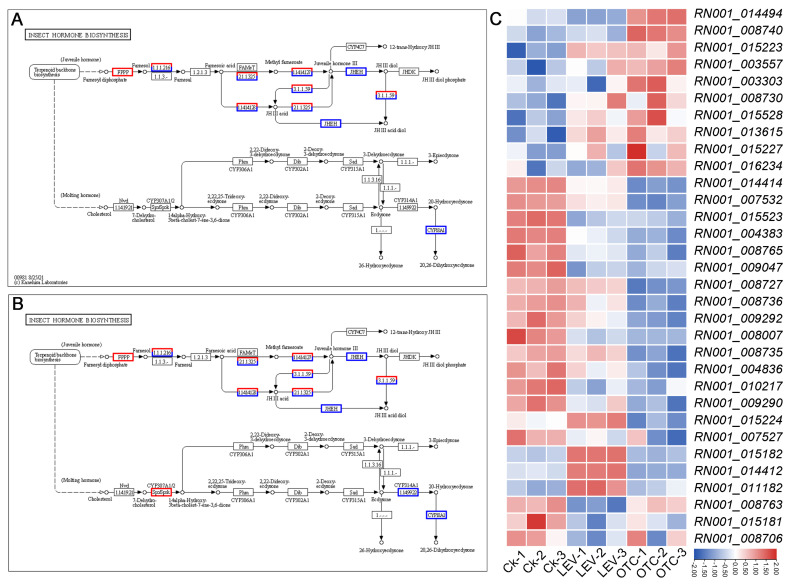
Dynamics of insect hormone biosynthesis and expression analyses of DEGs in *A. leii* among OTC and LEV treatments. (**A**) Dynamics of insect hormone biosynthesis in *A. leii* among LEV treatments. (**B**) Dynamics of insect hormone biosynthesis in *A. leii* among OTC treatments. (**C**) Expression analyses of 32 DEGs involved in insect hormone biosynthesis were performed based on the FPKM value.

To further investigate which genes are affected in the insect hormone biosynthesis pathway under OTC and LEV treatment conditions, we constructed a diagram of this pathway and performed expression analyses of DEGs. As shown in Figure 4, the expression levels of the juvenile hormone biosynthesis pathway within insect hormone biosynthesis were similarly affected under both the OTC and LEV treatments, indicating a possible conserved response mechanism to antibiotic treatments in *A. leii*. However, the molting hormone biosynthesis pathway was exclusively affected by the OTC treatment condition (Figure 4B). Specifically, the expression patterns of cytochrome P450 family 307 subfamily A (*CY307A1/2*; *RN001_016234*) were 2.48-fold upregulated, and expression of ecdysone 20-monooxygenase (*CYP314A1*; *RN001_004836*) was significantly decreased by 2.24-fold in the OTC treatment (Figure 4C).

### 3.5. Overall Metabolomic Structure of A. leii Under OTC and LEV Treatments

**Figure 5:**
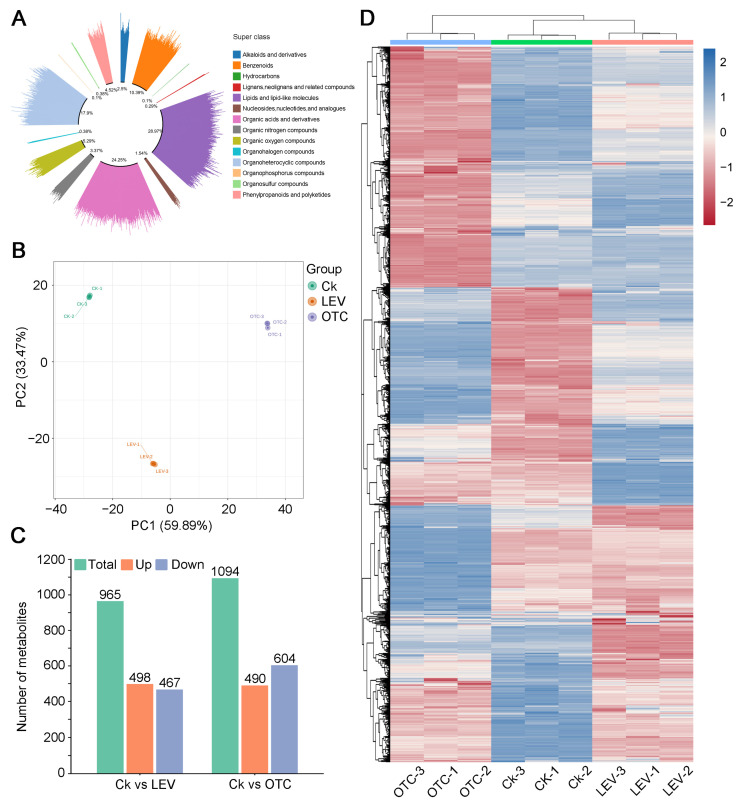
Metabolic alterations in the larvae of *A. leii* under OTC and LEV treatments. (**A**) Type and number of metabolites of the larvae of *A. leii* under OTC and LEV treatments. (**B**) The scatter score plot derived from PCA represents the metabolites of *A. leii* under the control, LEV, and OTC treatments. PCA scores depict the degree of metabolic profile separation between the three sample groups. Green, orange, and purple indicate the Ck, LEV, and OTC treatment groups, respectively. (**C**) Differentially expressed metabolites in *A. leii* under the LEV and OTC treatments. (**D**) Heatmap illustrating differentially expressed metabolites in *A. leii* in the Ck, LEV, and OTC treatment groups. Blue and red indicate upregulated and downregulated metabolites, respectively.

To explore shifts in metabolic pathways of *A. leii* in response to OTC and LEV treatments, metabolomic analysis was carried out using LC-MS to obtain a broad overview of metabolic changes. Metabolomic profiling revealed 2748 metabolites, which were categorized into 14 different biochemical classes (Figure 5A). Lipids and lipid-like molecules were the dominant compound class, constituting 28.97% of the total metabolites, followed by organic acids and derivatives (24.25%), organoheterocyclic compounds (17.90%), benzenoids (10.39%), and organic oxygen compounds (5.29%) (Figure 5A). PCA and statistical analysis of inter-sample correlation in metabolite abundance revealed distinct separations among metabolites and strong correlations between the biological replicates within the Ck, LEV, and OTC treatment groups (Figure 5B and Figure S2). A univariate statistical analysis via VIP scores from the OPLS-DA model indicated that 965 endogenous metabolites were impacted through LEV treatment (VIP >1.0, *p* < 0.05) (Figure 5C), where 498 and 467 metabolites were upregulated and downregulated, respectively. For the OTC treatment, 1094 endogenous metabolites were impacted (VIP > 1.0, *p* < 0.05) (Figure 5C), where 490 substances were upregulated, whereas 604 were downregulated. Additionally, the hierarchical clustering metabolomic heatmap clustering indicated pronounced shifts in metabolite levels between groups in *A. leii* under the OTC and LEV treatments (Figure 5D).

### 3.6. Metabolomic Profiles of A. leii Under OTC and LEV Treatments

**Figure 6:**
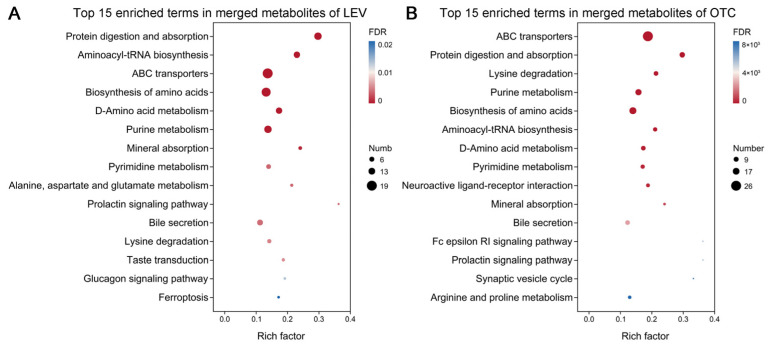
KEGG enrichment analysis of differential metabolites in *A. leii* under OTC and LEV treatments. (**A**) Top 15 enriched KEGG terms in merged metabolites of LEV. (**B**) Top 15 enriched KEGG terms in merged metabolites of OTC. Dots depict the quantity of metabolites enriched in the related KEGG pathway category. Color indicates the *p*-value, and the horizontal axis indicates the enrichment factor.

For exploring the functional pathways related to these altered metabolites, KEGG enrichment analysis was utilized. These differential metabolites were enriched in 136 KEGG pathways under the LEV treatment, where 53 were markedly enriched (*p* < 0.05). Notably, seven KEGG pathways, including “Protein digestion and absorption”, “Aminoacyl-tRNA biosynthesis”, “ABC transporters”, “Biosynthesis of amino acids”, “D-Amino acid metabolism”, “Purine metabolism”, and “Mineral absorption”, were most significantly enriched (FDR < 0.001) (Figure 6A and Table S7). For the OTC treatment, 144 KEGG pathways were found to associate, where 50 were markedly enriched (*p* < 0.05). Ten KEGG pathways, including “ABC transporters”, “Protein digestion and absorption”, “Lysine degradation”, “Purine metabolism”, “Biosynthesis of amino acids”, “Aminoacyl-tRNA biosynthesis”, “D-Amino acid metabolism”, “Pyrimidine metabolism”, “Neuroactive ligand-receptor interaction”, and “Mineral absorption”, were most significantly enriched (FDR < 0.001) (Figure 6B and Table S8). Collectively, these pathways, as revealed by the KEGG enrichment analysis, appear to be critically involved in the adaptive response of *A. leii* to the OTC and LEV treatments, particularly those pathways with an FDR of less than 0.001.

### 3.7. Integrated Transcriptome and Metabolome Analysis

**Figure 7:**
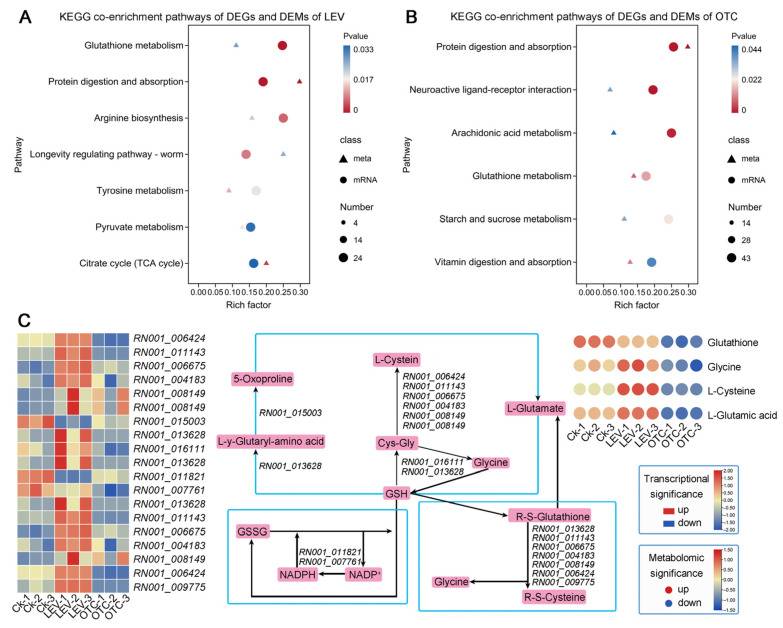
Correlation analysis of transcriptome and metabolomics data of the larvae of *A. leii* under OTC and LEV treatments. (**A**) KEGG co-enrichment analysis of transcriptome and metabolome of LEV treatment. (**B**) KEGG co-enrichment analysis of transcriptome and metabolome of OTC treatment. Triangles represent metabolites, and circles represent genes. Dots depict the quantity of metabolites enriched in the corresponding KEGG pathway category. Color indicates the *p*-value, and the horizontal axis indicates the enrichment factor. (**C**) Schematic diagram of glutathione metabolism under OTC and LEV treatments. Heatmaps of expression/abundance patterns of DEGs and DEMs.

To investigate the intricate interplay between DEGs and DAMs in *A. leii* under OTC and LEV treatments, we co-mapped DEGs and DAMs to the KEGG pathway framework, which offers enhanced resolution of gene–metabolite regulatory relationships. The results indicated that in the LEV group, the enriched pathways comprised “Glutathione metabolism”, “Protein digestion and absorption”, “Arginine biosynthesis”, “Longevity regulating pathway-worm”, and “Tyrosine metabolism” (Figure 7A). In the OTC group, the enriched pathways included “Protein digestion and absorption”, “Neuroactive ligand-receptor interaction”, “Arachidonic acid metabolism”, “Glutathione metabolism”, and “Starch and sucrose metabolism” (Figure 7B).

Based on the co-enrichment analysis of DEGs and DMAs, we concluded that glutathione metabolism played a key regulatory role related to *A. leii* under the OTC and LEV treatments. So, a glutathione metabolism pathway containing relevant metabolites and genes was drawn by integrating the KEGG database, and this pathway included fourDMAs and 19 DEGs (Figure 7C). Specifically, the expression levels of glutathione hydrolase (*RN001_013628*, *RN001_013628*, *RN001_013628*), aminopeptidase N (*RN001_011143*, *RN001_006675*, *RN001_004183*, *RN001_008149*), cytosol aminopeptidase (*RN001_009775*), leucyl aminopeptidase (*RN001_006424*), and glutathione-specific gamma-glutamylcyclotransferase (*RN001_016111*) were downregulated in *A. leii* under the OTC treatment. Likewise, the concentration levels of glutathione, glycine, L-cysteine, and L-glutamic acid were also downregulated in *A. leii* under the OTC treatment. Notably, the LEV induced upregulation of glutathione metabolism pathway-related genes and metabolites compared to the OTC treatment, implying that these changes might represent different coping strategies used by *A. leii* upon facing OTC and LEV treatments (Figure 7C).

## 4. Discussion

Oxytetracycline and levofloxacin are broad-spectrum antibiotics that have been widely used for many years for the clinical management of bacterial pathogens infecting humans and livestock [31,50]. However, up to 90% of ingested antibiotics are introduced into the environment via feces due to poor digestion or absorption, and they accumulate in both aquatic and terrestrial ecosystems [12,19,50]. At present, there are high concentrations of antibiotics in aquatic ecosystems, which can cause a series of adverse effects (e.g., oxidative stress) that adversely impact aquatic species [17,20,21,24,25]. Although several previous studies have demonstrated the possible effects of antibiotics on aquatic species, especially fish and shrimp, comprehensive research on the impact of antibiotics on aquatic insects, such as aquatic fireflies, remains insufficient [12,35,50]. The present work provides a systematic evaluation of long-term antibiotic exposure effects on *Aquatica leii* growth and development from transcriptional and metabolic perspectives.

Currently, the effects of antibiotics on weight gain of aquatic organisms lack consensus. For example, sulfamethoxazole and OTC were fed to zebrafish (*Danio rerio*), and the results indicated that OTC did not produce a growth-promoting effect on zebrafish, while body weight in the SMX-treated group was elevated compared to the controls [51]. Gentamicin sulfate and levofloxacin hydrochloride markedly inhibited *Musca domestica* larval development [52]. In comparison to controls, OTC- and LEV-treated groups showed notable increases in body length and body weight. Due to these inconsistent results, how antibiotics promote aquatic organism growth is an interesting question. Furthermore, previous studies have shown that antibiotics can lead to enhanced production of ROS in organisms, and ROS can further cause oxidative damage [12,53]. In response to these elevated ROS, SOD and CAT as the main antioxidant enzymes can resist excessive ROS attacks and maintain redox homeostasis [37,54]. Over extended OTC administration, the activities of SOD and CAT in *A. leii* larvae significantly increased (Figure 1C,D). During the long-term LEV treatment, the activities of SOD in *A. leii* larvae significantly decreased, while the activity of CAT showed no significant changes (Figure 1C,D). The present findings corroborate those of prior investigations on Nile tilapia (*Oreochromis niloticus*) chronically exposed to low environmental concentrations antibiotics demonstrating an upregulation of antioxidant enzyme activity under OTC treatment [12].

Recently, high-throughput transcriptome sequencing was employed for characterizing DEGs under stress conditions and exploring their involvement in various biological pathways [39,54]. Nonetheless, comprehensive transcriptomic investigations of *A. leii* are still scarce. So far, we only have information on the transcriptome related to benzo(a)pyrene stress, molecular adaptations to fresh water, and high-temperature tolerance traits for *A. leii* [7,55,56]. This research involved a comparative analysis of transcriptomic data to elucidate molecular responses to external stimuli of LEV- and OTC-treated *A. leii* larvae, as well as control larvae, to examine the response mechanisms of *A. leii* to antibiotic treatment. 1193 DEGs, including 679 up-regulated and 514 down-regulated genes, were detected in *A. leii* larvae with LEV treatment. Additionally, 1669 DEGs were identified under the OTC treatment, including 686 upregulated and 983 downregulated genes (Figure 2A). The observed transcriptomic shifts under the LEV and OTC treatments support previous evidence of extensive gene expression changes in organisms subjected to long-term exposure to environmentally relevant concentrations of antibiotics [52,57,58,59]. Furthermore, the markedly enriched KEGG terms were related to the digestive system, immune system, xenobiotics biodegradation and metabolism, metabolism of terpenoids and polyketides, signaling molecules and interaction, and aging, such as pancreatic secretion, protein digestion and absorption, longevity-regulating pathway—multiple species, insect hormone biosynthesis, glutathione metabolism, and neuroactive ligand–receptor interaction. Most of the enriched signaling pathways identified have previously been implicated in the antibiotic stress responses of other organisms, such as the saprophagous insect *Musca domestica* [52], zebrafish [59], and red seabream (*Pagrus major*) [60]. This analysis reveals that *A. leii* may utilize molecular pathways in response to antibiotics that are largely consistent with those observed in other animals. Insect metamorphosis development is co-regulated by the processes of synthesizing ecdysone and juvenile hormone [61]. The steroid hormone ecdysone drives molting and developmental transitions, whereas juvenile hormone inhibits these processes by opposing ecdysone, ensuring that metamorphosis does not occur prematurely [62,63]. In this study, 9 upregulated and 8 downregulated DEGs were observed in the juvenile hormone synthesis processes of the LEV group, while 10 up-regulated and 16 down-regulated DEGs were detected in the OTC group. It is interesting to note that we showed a substantial reduction in expression of juvenile-hormone-degrading enzymes, including juvenile hormone esterase (*RN001_008727*, *RN001_008736*, *RN001_009292*, *RN001_008007*, *RN001_008735*, *RN001_009290*) and juvenile hormone epoxide hydrolase (*RN001_010217*) (Figure 4). Previous studies have indicated that suppression of juvenile hormone levels, mediated by enhanced activity of JH-degrading enzymes, leads to the inhibition of reproductive and developmental processes [64]. In this study, the downregulation of JH-degrading enzymes led to increased JH levels, which may have promoted the growth and development of *A. leii* larvae. This finding is consistent with our measurements of body weight and length. However, without direct measurement of JH titers or enzyme activity, this link remains correlative and requires further functional validation.

Environmental adaptation is closely linked to dynamic changes in metabolic pathways such as stress [37,65]. Consistent metabolic signatures across diverse antibiotic treatments have been observed in a previous study [65]. In this study, the proportions of Amino acids, peptides, and analogues, glycerophosphocholines, fatty acids, and conjugates were higher than those of other metabolites in the LEV and OTC groups, and the ratios between these two groups were similar. These three types of DEMs are also the most prominent among the metabolic pathways annotated in KEGG. We hypothesize that they are key regulators of *A. leii* adaptation to the LEV and OTC treatments. L-Tyrosine is a non-essential aromatic amino acid involved in protein production and primarily supports the preparation of catecholamines, including dopamine, adrenaline, thyroid hormones, and melanin [66]. It is suggested that low contents of L-tyrosine can stimulate growth in animals [67]. In this study, L-tyrosine was upregulated 1.77-fold and 3.0-fold in *A. leii* under the LEV and OTC treatments, respectively, indicating that tyrosine biosynthesis was promoted under antibiotic treatment, thus facilitating larval growth and development. This is consistent with our body weight and length data. As a major cellular antioxidant, glutathione (GSH), synthesized from glutamic acid, cysteine, and glycine, mitigates oxidative stress and maintains redox homeostasis [68]. In this paper, levels of key metabolites in the glutathione metabolism pathway, such as glutathione, glycine, L-cysteine, and L-glutamic acid, were upregulated under the LEV treatment, whereas under the OTC treatment, they were downregulated (Figure 7). Meanwhile, the transcriptome analysis showed that the tendency toward DEGs expression was consistent with that of DEMs (Figure 7). The upregulation of the glutathione homeostasis pathway under the LEV treatment indicates that the detoxification process in the LEV group was active. This increase in the glutathione pathway could be a compensatory response to the downregulation of SOD under LEV treatment of *A. leii*. The same phenomenon appears in *Oncorhynchus mykiss* under acute heat stress as well [69]. Similarly, glutathione S-transferase (GST) activities in *Oncorhynchus mykiss* were significantly downregulated following OTC exposure [70]. For this paper, levels of key metabolites and genes in the glutathione metabolism pathway were downregulated. It is reported that exposure to oxytetracycline may induce the glutathione detoxification pathway, directly or indirectly involving potential metabolites, thereby leading to a reduction in detoxification capacity through GST involvement [70]. Therefore, OTC may induce detoxification mechanisms that consume large amounts of glutathione in *A.leii*, thereby leading to a decrease in glutathione levels. However, this interpretation remains hypothetical without direct biochemical evidence. Future studies must measure actual GST enzyme activities, quantify glutathione (GSH/GSSG) metabolite pools, and assess lipid peroxidation products (e.g., MDA) to validate this proposed compensatory mechanism.

Although our research has yielded some meaningful results, there are still several limitations. For example, the pooling strategy of combining 10 larvae per replicate to reduce individual variability represents a key limitation of our study. By doing so, we necessarily sacrifice the ability to make individual-level inferences and cannot capture inter-individual variation in transcriptional responses. An additional limitation is the lack of analytical verification of chemical concentrations in the exposure water. While we maintained nominal concentrations with daily solution renewal, the actual concentrations experienced by the larvae may have deviated from nominal values. This could affect the precision of our dose–response interpretations and the reproducibility of our findings. Future studies incorporating analytical chemistry would strengthen the reliability of exposure assessments. Furthermore, our transcriptome and metabolome results are largely inferential, and these mechanistic interpretations remain speculative and warrant validation through targeted biochemical and functional experiments.

## 5. Conclusions

In this study, the effects of low environmental concentrations of antibiotics on the growth and development in *A.leii* were investigated using an integrated transcriptional and metabolomic approach. Our findings indicate that *A. leii* larvae in the OTC and LEV treatment groups had significantly higher morphological indices (body length and weight) than the controls. The OTC and LEV treatments were able to facilitate substantial variations in SOD and CAT activities. The results revealed pronounced shifts in the larvae of *A. leii* following the OTC and LEV treatments. KEGG analyses indicated that ‘Insect hormone biosynthesis’ pathway-related genes were substantially modified under the OTC and LEV treatments. Metabolomics analyses showed that metabolites were found to be notably changed under the OTC and LEV treatments, and metabolic pathways associated with amino acids, organic acids, and carbohydrates were enhanced. The combined analysis indicated that glutathione metabolism was central to the molecular response of *A. leii* to the OTC and LEV treatments. The insights gained from this research can inform antibiotic management practices in *A. leii* aquaculture by clarifying tolerance mechanisms and appropriate dosing.

## Data Availability

Transcriptome analysis data have been published, and the NCBI accession number is PRJNA1372381. Metabolome raw data have been published, and the National Genomics Data Center (NGDC) accession number is PRJCA052586.

## Supplementary Materials

The following supporting information can be downloaded at: https://www.mdpi.com/article/10.3390/insects16121239/s1, Figure S1: Transcriptome Pearson’s correlation coefficient and principal component analysis under OTC and LEV treatments of *A. leii*. Figure S2: Heatmap of Pearson’s correlation coefficients for metabolite abundance between different samples within the Ck, LEV, and OTC treatment groups. Table S1: A comprehensive dataset reporting the identified compounds. Table S2: Effect of OTC and LEV treatments on body length and body weight of *Aquatica leii*. Table S3: SOD and CAT levels of *Aquatica leii* exposed to OTC and LEV treatments for a long time. Table S4: Summary of RNAsequencing of *Aquatica leii* exposed to OTC and LEV treatments for a long time. Table S5: Top 15 enriched KEGG pathways of DEGs under LEV treatment in *Aquatica leii*. Table S6: Top 15 enriched KEGG pathways of DEGs under OTC treatment in *Aquatica leii*. Table S7: Top 15 enriched KEGG terms in merged metabolites of LEV. Table S8: Top 15 enriched KEGG terms in merged metabolites of OTC.

## References

[1] Fu X., Zhu X. (2024). Key homeobox transcription factors regulate the development of the firefly’s adult light organ and bioluminescence. Nat. Commun..

[2] Lewis S.M., Cratsley C.K. (2008). Flash signal evolution, mate choice, and predation in fireflies. Annu. Rev. Entomol..

[3] Lewis S.M., Thancharoen A., Wong C.H., López-Palafox T., Santos P.V., Wu C., Reed J.M. (2021). Firefly tourism: Advancing a global phenomenon toward a brighter future. Conserv. Sci. Pract..

[4] Cheng S., Faidi M.A., Tan S.A., Vijayanathan J., Malek M.A., Bahashim B., Isa M.N.M. (2021). Fireflies in Southeast Asia: Knowledge gaps, entomotourism and conservation. Biodivers. Conserv..

[5] Lewis S.M., Jusoh W.F.A., Walker A.C., Fallon C.E., Joyce R., Yiu V. (2024). Illuminating firefly diversity: Trends, threats and conservation strategies. Insects.

[6] Pérez-Hernández C.X., Gutiérrez Mancillas A.M., Del-Val E., Mendoza-Cuenca L. (2023). Living on the edge: Urban fireflies (Coleoptera, Lampyridae) in Morelia, Michoacán, Mexico. Peerj.

[7] Liu C., Li J., Yan L., Zhu Y., Li Z., Cao C., Wang Y. (2025). Integrated mRNA and miRNA omics analyses reveal transcriptional regulation of the tolerance traits by *Aquatica leii* in response to high temperature. Insects.

[8] Szczepański M., Szajdak L.W., Meysner T. (2021). Impact of shelterbelt and peatland barriers on agricultural landscape groundwater: Carbon and nitrogen compounds removal efficiency. Agronomy.

[9] Lihan S., Lee S.Y., Toh S.C., Leong S.S. (2021). Plasmid-mediated antibiotic resistant *Escherichia coli* in Sarawak Rivers and aquaculture farms, Northwest of Borneo. Antibiotics.

[10] Al Salah D.M.M., Laffite A., Poté J. (2019). Occurrence of bacterial markers and antibiotic resistance genes in Sub-Saharan rivers receiving animal farm wastewaters. Sci. Rep..

[11] CDDEP The State of the World’s Antibiotics in 2021. https://onehealthtrust.org/wp-content/uploads/2021/02/SOWA_01.02.2021_Low-Res.pdf.

[12] Limbu S.M., Zhou L., Sun S.X., Zhang M.L., Du Z.Y. (2018). Chronic exposure to low environmental concentrations and legal aquaculture doses of antibiotics cause systemic adverse effects in Nile tilapia and provoke differential human health risk. Environ. Int..

[13] Sotto R.B., Medriano C.D., Cho Y., Kim H., Chung I.Y., Seok K.S., Song K.G., Hong S.W., Park Y., Kim S. (2017). Sub-lethal pharmaceutical hazard tracking in adult zebrafish using untargeted LC-MS environmental metabolomics. J. Hazard. Mater..

[14] Newton D.P., Ho P.Y., Huang K.C. (2023). Modulation of antibiotic effects on microbial communities by resource competition. Nat. Commun..

[15] Carvalho I.T., Santos L. (2016). Antibiotics in the aquatic environments: A review of the European scenario. Environ. Int..

[16] Kayani M.U.R., Yu K., Qiu Y., Shen Y., Gao C., Feng R., Zeng X., Wang W., Chen L., Su H.L. (2021). Environmental concentrations of antibiotics alter the zebrafish gut microbiome structure and potential functions. Environ. Pollut..

[17] Zhou L., Limbu S.M., Shen M., Zhai W., Qiao F., He A., Du Z.Y., Zhang M. (2018). Environmental concentrations of antibiotics impair zebrafish gut health. Environ. Pollut..

[18] Nakamura K., Takamatsu D., Harada M., Zendo T., Sekiya Y., Endo A. (2025). Nisin A treatment to protect honey bee larvae from european foulbrood disease. Probiotics Antimicrob. Proteins.

[19] Miao J., Yin Z., Yang Y., Liang Y., Xu X., Shi H. (2021). Abundance and dynamic distribution of antibiotic resistance genes in the environment surrounding a veterinary antibiotic manufacturing site. Antibiotics.

[20] Zhou J., Yun X., Wang J., Li Q., Wang Y. (2022). A review on the ecotoxicological effect of sulphonamides on aquatic organisms. Toxicol. Rep..

[21] Li Z., Lu T., Li M., Mortimer M., Guo L.H. (2023). Direct and gut microbiota-mediated toxicities of environmental antibiotics to fish and aquatic invertebrates. Chemosphere.

[22] González-González E.D., Gómez-Oliván L.M., Islas-Flores H., Galar-Martínez M. (2021). Developmental effects of amoxicillin at environmentally relevant concentration using zebrafish embryotoxicity test (ZET). Water Air Soil Poll..

[23] Lee S., Kim C., Liu X., Lee S., Kho Y., Kim W.K., Choi K. (2021). Ecological risk assessment of amoxicillin, enrofloxacin, and neomycin: Are their current levels in the freshwater environment safe?. Toxics.

[24] Lei Y., Li F., Mortimer M., Li Z., Peng B.X., Li M., Guo L.H., Zhuang G. (2023). Antibiotics disrupt lipid metabolism in zebrafish (*Danio rerio*) larvae and 3T3-L1 preadipocytes. Sci. Total Environ..

[25] Kim H.Y., Asselman J., Jeong T.Y., Yu S., De Schamphelaere K.A.C., Kim S.D. (2017). Multigenerational effects of the antibiotic Tetracycline on transcriptional responses of *Daphnia magna* and its relationship to higher levels of biological organizations. Environ. Sci. Technol..

[26] Zhang Q., Cheng J., Xin Q. (2015). Effects of tetracycline on developmental toxicity and molecular responses in zebrafish (*Danio rerio*) embryos. Ecotoxicology.

[27] Schwarz C., Procaccianti C., Mignot B., Sadafi H., Schwenck N., Murgia X., Bianco F. (2021). Deposition of inhaled Levofloxacin in cystic fibrosis lungs assessed by functional respiratory imaging. Pharmaceutics.

[28] Zhou Z., Zhang Z., Feng L., Zhang J., Li Y., Lu T., Qian H. (2020). Adverse effects of levofloxacin and oxytetracycline on aquatic microbial communities. Sci. Total Environ..

[29] Dong D., Zhang L., Liu S., Guo Z., Hua X. (2016). Antibiotics in water and sediments from Liao River in Jilin Province, China: Occurrence, distribution, and risk assessment. Environ. Earth Sci..

[30] Chen H., Liu S., Xu X.R., Zhou G.J., Liu S.S., Yue W.Z., Ying G.G. (2015). Antibiotics in the coastal environment of the Hailing Bay region, South China Sea: Spatial distribution, source analysis and ecological risks. Mar. Pollut. Bull..

[31] Wu Y., Yu W., Song Z., He J., Li Z., Chen Q., Wang S., Li P., Cheng S. (2025). The Acute Toxicity and Cardiotoxic Effects of Levofloxacin on Zebrafish (*Danio rerio*). Toxics.

[32] Van Boeckel T.P., Gandra S., Ashok A., Caudron Q., Grenfell B.T., Levin S.A., Laxminarayan R. (2014). Global antibiotic consumption 2000 to 2010: An analysis of national pharmaceutical sales data. Lancet Infect. Dis..

[33] Li J., Li W., Liu K., Guo Y., Ding C., Han J., Li P. (2022). Global review of macrolide antibiotics in the aquatic environment: Sources, occurrence, fate, ecotoxicity, and risk assessment. J. Hazard. Mater..

[34] Yang J., Ahmed W., Mehmood S., Ou W., Li J., Xu W., Wang L., Mahmood M., Li W. (2023). Evaluating the combined effects of Erythromycin and Levofloxacin on the growth of *Navicula* sp. and understanding the underlying mechanisms. Plants.

[35] Sun S., Korheina D.K.A., Fu H., Ge X. (2020). Chronic exposure to dietary antibiotics affects intestinal health and antibiotic resistance gene abundance in oriental river prawn (*Macrobrachium nipponense*), and provokes human health risk. Sci. Total Environ..

[36] Hanna N., Tamhankar A.J., Stålsby Lundborg C. (2023). Antibiotic concentrations and antibiotic resistance in aquatic environments of the WHO Western Pacific and South–East Asia regions: A systematic review and probabilistic environmental hazard assessment. Lancet Planet. Health.

[37] Wang Z., Li J., Zhao P., Yu Z., Yang L., Ding X., Lv H., Yi S., Sheng Q., Zhang L. (2024). Integrated microbiome and metabolome analyses reveal the effects of low pH on intestinal health and homeostasis of crayfish (*Procambarus clarkii*). Aquat. Toxicol..

[38] Chen S., Zhou Y., Chen Y., Gu J. (2018). fastp: An ultra-fast all-in-one FASTQ preprocessor. Bioinformatics.

[39] Zhou F., Qi M., Li J., Huang Y., Chen X., Liu W., Yao G., Meng Q., Zheng T., Wang Z. (2023). Comparative transcriptomic analysis of Largemouth Bass (*Micropterus salmoides*) livers reveals response mechanisms to high temperatures. Genes.

[40] Kim D., Langmead B., Salzberg S.L. (2015). HISAT: A fast spliced aligner with low memory requirements. Nat. Methods.

[41] Pertea M., Pertea G.M., Antonescu C.M., Chang T.C., Mendell J.T., Salzberg S.L. (2015). StringTie enables improved reconstruction of a transcriptome from RNA-seq reads. Nat. Biotechnol..

[42] Anders S., Pyl P.T., Huber W. (2015). HTSeq—A Python framework to work with high-throughput sequencing data. Bioinformatics.

[43] Li B., Dewey C.N. (2011). RSEM: Accurate transcript quantification from RNA-Seq data with or without a reference genome. BMC Bioinform..

[44] Love M.I., Huber W., Anders S. (2014). Moderated estimation of fold change and dispersion for RNA-seq data with DESeq2. Genome Biol..

[45] Wu T., Hu E., Xu S., Chen M., Guo P., Dai Z., Feng T., Zhou L., Tang W., Zhan L. (2021). clusterProfiler 4.0: A universal enrichment tool for interpreting omics data. Innovation.

[46] Want E.J., Masson P., Michopoulos F., Wilson I.D., Theodoridis G., Plumb R.S., Shockcor J., Loftus N., Holmes E., Nicholson J.K. (2013). Global metabolic profiling of animal and human tissues via UPLC-MS. Nat. Protoc..

[47] Alseekh S., Aharoni A., Brotman Y., Contrepois K., D’Auria J., Ewald J., Ewald J.C., Fraser P.D., Giavalisco P., Hall R.D. (2021). Mass spectrometry-based metabolomics: A guide for annotation, quantification and best reporting practices. Nat. Methods.

[48] Tsugawa H., Cajka T., Kind T., Ma Y., Higgins B., Ikeda K., Kanazawa M., VanderGheynst J., Fiehn O., Arita M. (2015). MS-DIAL: Data-independent MS/MS deconvolution for comprehensive metabolome analysis. Nat. Methods.

[49] Qian G., Wang M., Wang X., Liu K., Li Y., Bu Y., Li L. (2023). Integrated transcriptome and metabolome analysis of Rice leaves response to high Saline-Alkali stress. Int. J. Mol. Sci..

[50] Griboff J., Carrizo J.C., Bacchetta C., Rossi A., Wunderlin D.A., Cazenave J., Amé M.V. (2025). Effects of short-term dietary oxytetracycline treatment in the farmed fish *Piaractus mesopotamicus*. Environ. Toxicol. Chem..

[51] Zhou L., Limbu S.M., Qiao F., Du Z.Y., Zhang M. (2018). Influence of long-term feeding antibiotics on the gut health of Zebrafish. Zebrafish.

[52] Li T., Zhang Q., Zhang X., Wan Q., Wang S., Zhang R., Zhang Z. (2021). Transcriptome and microbiome analyses of the mechanisms underlying antibiotic-mediated inhibition of larval development of the saprophagous insect *Musca domestica* (Diptera: Muscidae). Ecotoxicol. Environ. Safe..

[53] Li G., Xia X., Zhao S., Shi M., Liu F., Zhu Y. (2020). The physiological and toxicological effects of antibiotics on an interspecies insect model. Chemosphere.

[54] Wang Z., Yang L., Zhou F., Li J., Wu X., Zhong X., Lv H., Yi S., Gao Q., Yang Z. (2023). Integrated comparative transcriptome and weighted gene co-expression network analysis provide valuable insights into the response mechanisms of crayfi38sh (*Procambarus clarkii*) to copper stress. J. Hazard. Mater..

[55] Zhang Q.L., Li H.W., Dong Z.X., Yang X.J., Lin L.B., Chen J.Y., Yuan M.L. (2020). Comparative transcriptomic analysis of fireflies (Coleoptera: Lampyridae) to explore the molecular adaptations to fresh water. Mol. Ecol..

[56] Zhang Q.L., Guo J., Deng X.Y., Wang F., Chen J.Y., Lin L.B. (2019). Comparative transcriptomic analysis provides insights into the response to the benzo(a)pyrene stress in aquatic firefly (*Luciola leii*). Sci. Total Environ..

[57] Liu Q., Jin Z., Liao X., Feng J., Li Z., Zhao W., Liu H. (2025). Nano-Silicon mitigates Sulfamethoxazole-induced stress in Wheat seedlings: Integrated transcriptomic and metabolomic analysis. J. Environ. Chem. Eng..

[58] Xin R., Yu X., Fan J. (2022). Physiological, biochemical and transcriptional responses of cyanobacteria to environmentally relevant concentrations of a typical antibiotic-roxithromycin. Sci. Total Environ..

[59] Zhang G., Xu Y., Xia Y., Wang G., Zhao H. (2022). Transcriptomic analysis of hepatotoxicology of adult Zebrafish (*Danio rerio*) exposed to environmentally relevant Oxytetracycline. Arch. Environ. Contam. Toxicol..

[60] Iida M., Nguyen H.T., Takahashi F., Bak S.M., Kanda K., Iwata H. (2022). Effects of exposure to oxytetracycline on the liver proteome of red seabream (*Pagrus major*) in a real administration scenario. Comp. Biochem. Physiol. C Toxicol. Pharmacol..

[61] Hiruma K., Kaneko Y. (2013). Hormonal regulation of insect metamorphosis with special reference to juvenile hormone biosynthesis. Curr. Top. Dev. Biol..

[62] Liu S., Li K., Gao Y., Liu X., Chen W., Ge W., Feng Q., Palli S.R., Li S. (2018). Antagonistic actions of juvenile hormone and 20-hydroxyecdysone within the ring gland determine developmental transitions in *Drosophila*. Proc. Natl. Acad. Sci. USA.

[63] Lei Y., Guo J., Chen Q., Mo J., Tian Y., Iwata H., Song J. (2022). Transcriptomic alterations in Water Flea (*Daphnia magna*) following pravastatin treatments: Insect hormone biosynthesis and energy metabolism. Toxics.

[64] Izadi H. (2025). Endocrine and enzymatic shifts during insect diapause: A review of regulatory mechanisms. Front. Physiol..

[65] Zampieri M., Zimmermann M., Claassen M., Sauer U. (2017). Nontargeted metabolomics reveals the multilevel response to antibiotic perturbations. Cell Rep..

[66] Slominski A., Zmijewski M.A., Pawelek J. (2012). L-tyrosine and L-dihydroxyphenylalanine as hormone-like regulators of melanocyte functions. Pigm. Cell Melanoma Res..

[67] Uehara A., Maekawa M., Nakagawa K. (2025). L-Tyrosine enhances tight junction integrity and anti-inflammatory properties: A potential alternative to antibiotic growth promoters in broilers. Arch. Microbiol..

[68] Islam M.M., Ahmed S.T., Kim Y.J., Mun H.S., Kim Y.J., Yang C.J. (2014). Effect of sea tangle (*Laminaria japonica*) and charcoal supplementation as alternatives to antibiotics on growth performance and meat quality of Ducks. Asian Australas. J. Anim. Sci..

[69] Zhou C., Yang S., Ka W., Gao P., Li Y., Long R., Wang J. (2022). Association of gut microbiota with metabolism in Rainbow Trout under acute heat stress. Front. Microbiol..

[70] Rodrigues S., Antunes S.C., Correia A.T., Nunes B. (2018). Oxytetracycline effects in specific biochemical pathways of detoxification, neurotransmission and energy production in *Oncorhynchus mykiss*. Ecotoxicol. Environ. Saf..

