# Supplementary material for: Unraveling the Impacts of Long-Term Exposure in Low Environmental Concentrations of Antibiotics on the Growth and Development of Aquatica leii (Coleoptera: Lampyridae) from Transcription and Metabolism
Source: Insects. 2025 Dec 8;16(12):1239. doi: 10.3390/insects16121239 (PMC12733991; doi:10.3390/insects16121239)
Supplement: Supplementary file 1 [file insects-16-01239-s001.zip › Supplementary Figures.pdf]

Figure S1

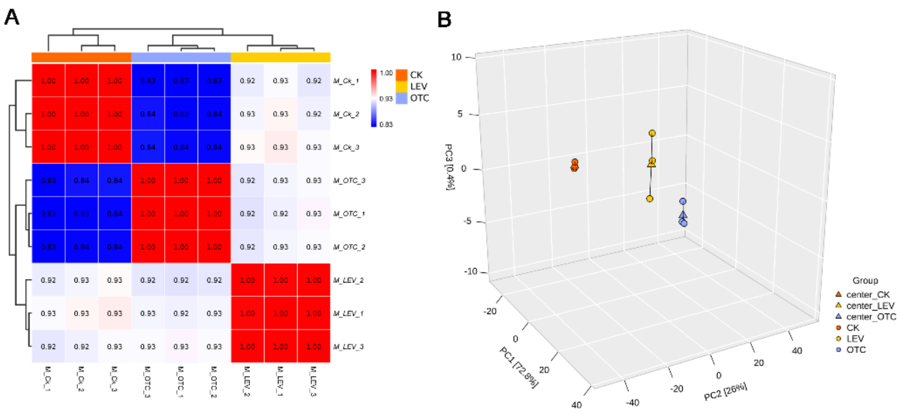

Figure S1. Transcriptome pearson correlation coefficient and principal component analysis under OTC and LEV treatments of *A. leii*. (A) Pearson correlation coefficient of transcriptome samples. Higher values indicate stronger correlation. (B) Principal component analysis plot. Circles indicate distinct sequencing samples, and triangles indicate the average values.

Figure S2

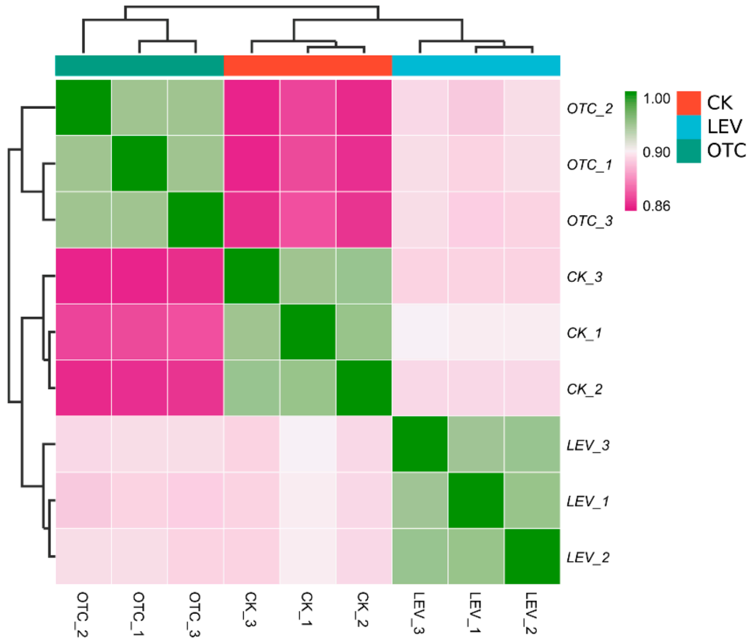

Figure S2. Heatmap of Pearson correlation coefficients for metabolite abundance between different samples within the Ck, LEV, and OTC treatment groups.
